# Simpson-Golabi-Behmel syndrome in one of the Dichorionic-diamniotic twin: a case report and literature review

**DOI:** 10.1186/s12884-021-04309-z

**Published:** 2022-01-17

**Authors:** Yongbing Guo, Huijing Zhang, Lixin Fan, Junya Chen, Xiaoxiao Zhang, Huixia Yang, Yu Sun

**Affiliations:** Department of Obstetrics and Gynecology, Peking University First Hospital, Beijing, 100034 China

**Keywords:** Simpson-Golabi-Behmel syndrome, Dichorionic-diamniotic twin, Fetal medicine, Case report

## Abstract

**Background:**

Simpson-Golabi-Behmel syndrome (SGBS) is a rare X-linked overgrowth syndrome. The main clinical manifestations are overgrowth and multiple malformations.

**Case presentation:**

A 38-year-old Chinese woman was pregnant with dichorionic-diamniotic (DCDA) twins after in-vitro fertilization. Series of ultrasound examinations indicated that the measurements (abdominal circumference and estimated foetal weight) of one twin were significantly greater than those of the other one. The genetic testing results of the larger baby indicated of Simpson-Golabi-Behmel syndrome.

**Conclusion:**

SGBS is difficult to diagnose due to different clinical manifestations. Clinicians need to be more aware of typical SGBS’s clinical findings and choose genetic testing methods individually to improve its prenatal diagnosis.

## Background

Simpson-Golabi-Behmel syndrome (SGBS) (OMIM: 312,870; ORPHAn373) is a rare X-linked overgrowth syndrome. This disease was first reported by Joe Leigh Simpson et al. in 1975 [1] and subsequently by Golabi and Rosen [2] as well as Behmel [3] in 1984. In 1988, Neri summarized previous case reports and named the disease as SGBS [4]. SGBS is clinically classified into 2 types: SGBS type I (OMIM: 312,870; ORPHAn373) and SGBS type II (OMIM 300,209; ORPHA79022). Between the 2 types, SGBS type I is more common[1–3] and is mainly associated with the mutation of GPC3, a gene located on Xp26 that codes for glypican-3. The main manifestations of type I include foetal overgrowth, polyhydramnios, craniofacial abnormalities, organ enlargement, limb abnormalities, cardiac structural abnormalities, digestive system abnormalities, genitourinary system abnormalities and bone abnormalities [5]. SGBS type II was firstly described by Terespolsky et al. in 1995, which is often associated with foetal hydrops and multiple malformations. In the case report, three out of four males (one was terminated at 19 gestational weeks) were hydropic at birth and died within 8 weeks of life due to multiple complications. The causes of death included pneumonia with meconium aspiration and sepsis (case IV-2), necrotizing enterocolitis, disseminated intravascular coagulopathy, secondary Klebsiella sepsis and acute renal failure (case IV-5), pneumonia and sepsis (case. IV-7). Therefore, it is fatal postnatally [6]. Type II is caused by mutation of the PIGA gene located on Xp22 [7, 8]. Therefore, genotyping is required for distinguishing the 2 types. As an X chromosome recessive disorder, most male paediatric patients will have more severe symptoms and will die of congenital heart disease or sepsis in the neonatal period, while female gene carriers can survive to adulthood, according to Mendel’s laws of inheritance. The life expectancy varies from person to person, which is related to specific gene mutations [9].

## Case presentation

**Fig. 1:**
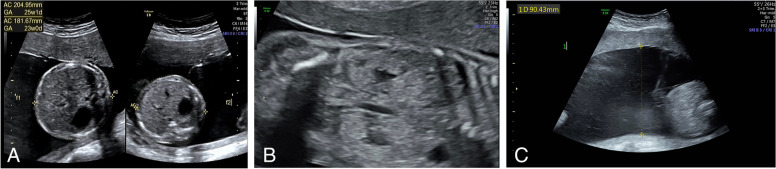
Ultrasound images at 22 + 2 gestational weeks. A, comparison of AC measurements between two foetuses (left: fetus A; Right: fetus B). B, hyperechoic renal cortex in foetus A. C, polyhydramnios in foetus A

**Fig. 2:**
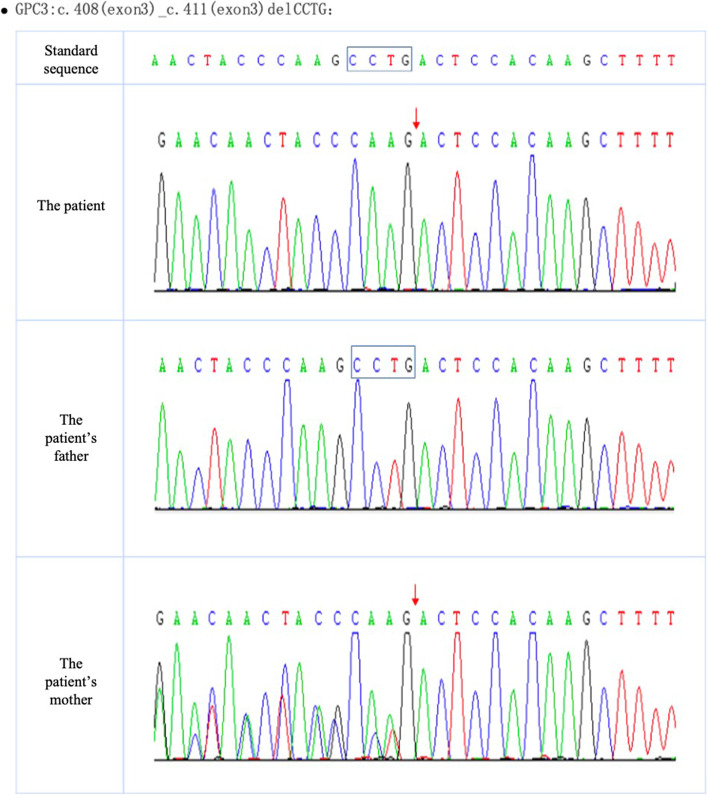
Gene testing report of the affected baby and his parents

A 38-year-old Chinese woman with Gravida 0 and Parity 0, claimed no history or family history of chronic disease. The pregnancy was conceived by assisted reproductive technology, and she received routine prenatal care at Peking University First Hospital, China. Ultrasound at 12 weeks of gestation indicated a dichorionic-diamniotic (DCDA) twin pregnancy: foetus A’s nuchal translucency (NT) was 2.36 mm and crown–rump length (CRL)was 64.5 mm; foetus B’s NT was 0.72 mm and CRL was 58.3 mm. The pregnant woman chose to undergo non-invasive prenatal testing (NIPT), the results of which showed low risk. At 19^+1^ weeks of gestation, sonographic biometrics revealed that foetus A was larger than expected, while foetus B showed no obvious abnormalities. At 22^+2^ weeks of gestation, Anomaly scan indicated that Foetus A’s abdominal circumference (AC) and estimated foetal weight (EFW) were both greater than the 97^th^ percentile [10], complicated with polyhydramnios and hyperechoic kidneys (Fig. 1), while foetus B followed the normal growth curve with no abnormal findings. Amniocentesis was then performed for the Foetus A at 23 weeks of gestation. The karyotype and array-based comparative genomic hybridization (aCGH) were unremarkable. Meantime, the cervix was measured 22.1 mm. Therefore, the pregnant woman was admitted to the hospital to undergo cervical cerclage due to short cervix at 24 weeks of gestation. Three days after the cerclage, membrane of Foetus A ruptured. After tocolytic, anti-inflammatory and lung-maturing treatments, no more vaginal leaking was observed. Afterwards, series of ultrasound examinations still indicated that the measurements of foetus A remained to be more than 90^th^ centile (Table 1) according to the fetal growth standrds for Chinese twin pregnancies [10], complicated with hyperechoic renal cortex and polyhydramnios. At 30 weeks of gestation, foetal echocardiography revealed that foetus A had a first-degree atrioventricular block. The pregnant woman and her family members requested to continue the pregnancy. At 31^+5^ weeks of gestation, forceps delivery was performed due to "foetal distress and intrauterine infection". The first male baby weighed 2250 g with an Apgar score 1 min and 5 min of 7 and 8; the second male baby weighed 1550 g with an Apgar score 1 min and 5 min of 9 and 9. Both foetuses were transferred to the new-born intensive care unit (NICU) for due to prematurity. In the NICU, the first newborn was significantly larger than the normal reference at this gestation age, with manifestations of abnormal appearance, stubby toes, gastric perforation, a ventricular septal defect (1.8 mm), hypertrophic pyloric stenosis, accessory breast, cryptorchidism, and hydrocele. A laparotomic gastric perforation repair and advanced genetic testing were performed on the 7^th^ day after birth. The genetic testing results returned with ChrX: 132,888,130–132,888,133: c.408-c.411delCCTG (Fig. 2), indicative of Simpson-Golabi-Behmel syndrome. The paediatric patient was treated in the NICU for 51 days after birth, and her family requested discharge from the hospital strongly, despite of severe sepsis and difficulties.

**Table 1:** Biometric measurements of the twin

Gestational Weeks	Fetus A						Fetus B					
	BPD(mm)	HC(mm)	AC(mm)	FL(mm)	SDP^a^(mm)	EFW(g)	BPD(mm)	HC(mm)	AC(mm)	FL(mm)	SDP(mm)	EFW(g)
19^+1^w	48.6 (> 97^th^)	170.6 (97^th^)	164.8 (> 97^th^)	31.7 (97^th^)	68	364 (> 97^th^)	44.3 (50^th^)	158.9 (50^th^)	137.8 (50^th^)	30 (90^th^)	33	275 (50^th^)
22^+2^w	60.0 (> 97^th^)	206.8 (97^th^)	205.0 (> 97^th^)	42.5 (> 97^th^)	85	680 (> 97^th^)	55.5 (75^th^)	196.4 (50^th^)	181.7 (90^th^)	37.6 (50^th^)	48	505 (75^th^)
26^+1^w	70.7 (90^th^)	251.5 (95^th^)	249.8 (> 97^th^)	50.4 (90^th^)	91	1189 (> 97^th^)	68.7 (75^th^)	244.9 (75^th^)	216.1 (50^th^)	48.7 (75^th^)	36	914 (75^th^)
28^+1^w	77.3 (95^th^)	275.9 (95^th^)	266.4 (> 97^th^)	56.7 (97^th^)	90	1554 (> 97^th^)	72.9 (50^th^)	259.1 (50^th^)	238.6 (50^th^)	52.7 (75^th^)	51	1178 (75^th^)
29^+2^w	79.0 (95^th^)	285.3 (97^th^)	281.6 (> 97^th^)	58.4 (95^th^)	93	1784 (> 97^th^)	76.7 (75^th^)	266.1 (50^th^)	245.2 (50^th^)	54.9 (75^th^)	50	1298 (50^th^)
31^+2^w	84.5 (> 97^th^)	303.6 (97^th^)	301.8 (> 97^th^)	61.0 (90^th^)	85	2170 \(> 97^th^)	79.8 (50^th^)	284.9 (50^th^)	256.7 (25^th^)	59.5 (75^th^)	40	1575 (50^th^)

^a^*SDP* single deepest pocket

## Discussion and conclusion

SGBS is difficult to diagnose due to different clinical manifestations. In this case, the biometric measurements of one twin were significantly larger than that of the other since the second trimester (19 weeks of gestation) (Table 1). In general, foetal macrosomia (namely, estimated weight of the foetus is greater than the 90^th^ percentile) is associated with gestation week calculation errors, maternal comorbidities (such as advanced age, obesity, pregestational diabetes, and gestational diabetes), and previous history of macrosomia. However, in addition to the above common situations, it is necessary to further consider overgrowth-related syndromes, which mainly include Beckwith-Wiedemann syndrome (BWS; OMIM 130,650), SGBS, Sotos syndrome, Weaver syndrome, and Pearlman syndrome. Although these syndromes have some overlapping clinical manifestations, such as excessive growth and polyhydramnios, they have their own characteristics, such as macroglossia and omphalocele in BWS; long head deformity and advanced bone age in Sotos syndrome and SGBS; micrognathia in Weaver syndrome; and renal enlargement and an inverted V-shaped upper lip in Pearlman syndrome. Of course, the final definitive diagnosis requires genetic testing [11].

The affected foetus in this case presented foetal overgrowth, polyhydramnios, along with [12] hyperechoic renal cortex and arrhythmia (first-degree atrioventricular block) during the pregnancy; therefore, our centre provided the patient with the option of amniocentesis. The patient agreed, but the ensuing karyotype and array comparative genomic hybridization (aCGH) results showed no abnormalities. Gastric perforation (pathologically diagnosed as a congenital gastric wall muscle defect) was detected on the 7^th^ day after birth, and other manifestations such as a ventricular septal defect (1.8 mm), hypertrophic pyloric stenosis, accessory breast, cryptorchidism, and hydrocele were also discovered postnatally, which are primarily consistent with the specific signs summarized by Cottereau et al. in their review, The specific signs include craniofacial abnormalities (macrocephaly, coarse/square face, hypertelorism eyes, cleft lip and palate, etc.), organomegaly (macroglossia, nephromegaly, hepatomegaly, tumour, etc.), limb abnormalities (broad finger, brachydactyly, postaxial polydactyly, etc.), cardiac structural abnormalities (structural defects and arrhythmia), digestive system abnormalities (diaphragmatic hernia, etc.), genitourinary system abnormalities (renal dysplasia, enhanced renal cortical echoes, renal cyst, cryptorchidism, hydrocele, etc.), skeletal abnormalities (scoliosis, thoracic abnormalities, etc.), and accessory breast, etc. [5]. Hence, a meticulous and accurate prenatal imaging examination will provide an important basis for diagnosis and for the selection of prenatal diagnostic techniques.

Additionally, the karyotype and aCGH result during pregnancy may not be able to diagnose SGBS definitively, especially in the absence of a proband. Kehrer et al. reported that a paediatric patient’s amniotic fluid results showed no abnormalities prenatally, and that frameshift mutations in GPC3 were found by postnatal whole-exome sequencing (WES). On the other hand, when proposita is identified, the deletion could be detected from fetal DNA by aCGH [12]. To note, current guidelines of neither the American College of Obstetricians and Gynaecologists (ACOG) or the American Society of Maternal–Fetal Medicine (SMFM) recommend the impletation of WES in routine clinical prenatal diagnosis [13]. However, traditional karyotyping and microarray analysis can only detect up to 40% of foetal genetic abnormalities [14]. A prospective cohort study of the application of WES for prenatal diagnosis by Petrovski et al. showed that WES could detect an additional 20% of significant gene mutations for fetal structural abnormalities with normal traditional karyotype and aCGH results [15], thereby further increasing the detection rate of genetic diseases. The NHS England (Genomics) is developing service infrastructure for prenatal exome sequencing if a fetus with multiple anomalies and selected other abnormalities are detected on foetal imaging. The selected othere abnormalities includeds skeletal dysplasias, large echogenic kidneys with a normal bladder, majar CNS abnormalities, and multiple contractures, etc. [16].

In our case, peripheral blood of the paediatric patient and his parents was subjected to trio whole-exome sequencing and copy number variation sequencing (trioWES + trioCNVseq), revealing a CCTG deletion at the c.408-c.411 site in exon 3 of the GPC3 gene in the child. The child was hemizygous, and his mother was heterozygous, which is consistent with the pathogenesis of X chromosome recessive (XR) disease (Fig. 2). GPC3 is located at Xq 26.2 and includes 8 exons with a total length of 2.3 kb (NCBI reference sequence NM_004484.3) [17]. To date, there are 86 different mutations, of which 34.9% are deletion mutations, 16.3% are nonsense mutations, 8.1% are missense mutations, 8.1% are duplication mutations, 4.7% are splicing site mutations, 2.3% are translocation mutations, and 1.2% are codon mutations [18]. In this case, the paediatric patient had a frameshift mutation caused by the deletion of the nucleotides CCTG, prematurely terminating protein synthesis and affecting the normal function of the protein.

## Data Availability

The datasets obtained and/or analyzed during the current study are available. From the corresponding author on reasonable request.

## Abbreviations

SGBS: Simpson-Golabi-Behmel syndrome
DCDA: Dichorionic-diamniotic
NT: Nuchal translucency
CRL: Crown–rump length
NIPT: Non-invasive prenatal testing
AC: Abdominal circumference
EFW: Estimated foetal weight
aCGH: Array comparative genomic hybridization
NICU: New-born intensive care unit
WES: Whole-exome sequencing
ACOG: American College of Obstetricians and Gynaecologists
SMFM: Society of Maternal–Fetal Medicine
XR: X chromosome recessive
BWS: Beckwith-Wiedemann syndrome
